# Tobacco Use Worldwide: Legislative Efforts to Curb Consumption

**DOI:** 10.29024/aogh.2362

**Published:** 2018-11-05

**Authors:** M. Teresa Perez-Warnisher, M. Pilar Carballosa de Miguel, Luis M. Seijo

**Affiliations:** 1 Clínica Universidad de Navarra, Madrid, ES; 2 Fundación Jimenez Díaz, Madrid, ES; 3 Ciberes, ES

## Abstract

Tobacco smoking is recognized as a major preventable cause of disease worldwide and is linked to 6 million deaths annually, 30% of which are due to cancer. The negative health consequences of smoking currently represent one of the greatest public health challenges. Secondhand smoke, declared carcinogenic by the International Agency for Research on Cancer in 2004, is also a major source of morbidity and premature death in nonsmokers, particularly children. Negative health effects associated with exposure to secondhand smoke have been well documented and include lung cancer, cardiovascular disease, asthma, and other respiratory diseases. International and national policies to implement cost-effective strategies to curtail smoking will have a significant impact on population health and will protect nonsmokers. Effective interventions, such as a combination of smoke-free laws, tobacco price increases, easy access to tobacco cessation treatments, and anti-tobacco media campaigns, should continue. Reducing tobacco use would be a major step towards the goal of decreasing health disparities by 2030 as 80% of the projected tobacco-related deaths will occur in low- and middle-income countries.

## Introduction

Tobacco use remains a major public health concern. Cigarette smoking has been linked to countless illnesses, chief among them cancer and cardiovascular and respiratory diseases, and is the single most preventable cause of death worldwide [1,2,3,4,5,6]. As many as 9% of all deaths are attributable to tobacco consumption, making smoking cessation the most cost-effective strategy for reducing morbidity and mortality [7,8,9]. According to the World Health Organization (WHO), tobacco kills nearly 6 million people every year worldwide, more than the human immunodeficiency virus (HIV), tuberculosis, and malaria combined. Unfortunately, despite recent favorable trends in developed nations, more than 8 million people are expected to die every year by 2030 [10].

Tobacco smoking has spread globally, currently increasing in many low- and middle-income countries. It is slowly but steadily decreasing in several high-income countries [11]. Paradoxically, death rates due to smoking-related illness are lower in low- than in middle- and high-income countries because of the delayed effects of cigarette smoking on health outcomes. Mortality in low- and middle-income countries, especially for women, will therefore continue to rise in the foreseeable future, even if efforts to reduce smoking are successful [11].

The reduction in smoking prevalence that has taken place over the past half century in most industrialized nations has not been evenly distributed among all groups of smokers. In particular, young adults, disadvantaged individuals, and women have experienced proportionally smaller declines [12,13]. Many factors influence smoking trends, including individual-level variables such as socioeconomic status and education, as well as system-level factors such as regional economic development and tobacco control policies [14,15]. Sociodemographic vulnerabilities may provide important clues for improving policy initiatives for tobacco control and regulation [16].

## Smoking Prevalence Around the World

Worldwide, approximately 23% of adults, including more than 1 billion males and 250 million females, smoke tobacco products. This gender gap is narrowing as the number of female smokers has been increasing. Unfortunately, smoking prevalence tends to be highest among those with the lowest levels of education and income. Nearly 80% of the world’s smokers live in low- and middle-income countries [17,18]. Current projections indicate that the number of smokers will increase globally to 1.6 billion over the next 25 years. As a consequence, the number of tobacco-related deaths will surpass the combined mortality from AIDS, tuberculosis, automobile accidents, maternal deaths, homicide, and suicide [19].

Consumption of tobacco products is increasing worldwide but unevenly; although it is decreasing in some high- and upper middle-income countries, it is markedly increasing in developing regions [20]. This pattern reflects the commercial strategy of tobacco companies; as smoking becomes less acceptable and profitable in the developed world, countries with fewer public health warnings and fewer restrictions on the commercialization of tobacco products are targeted.

## Global Tobacco Burden

**Figure 1:**
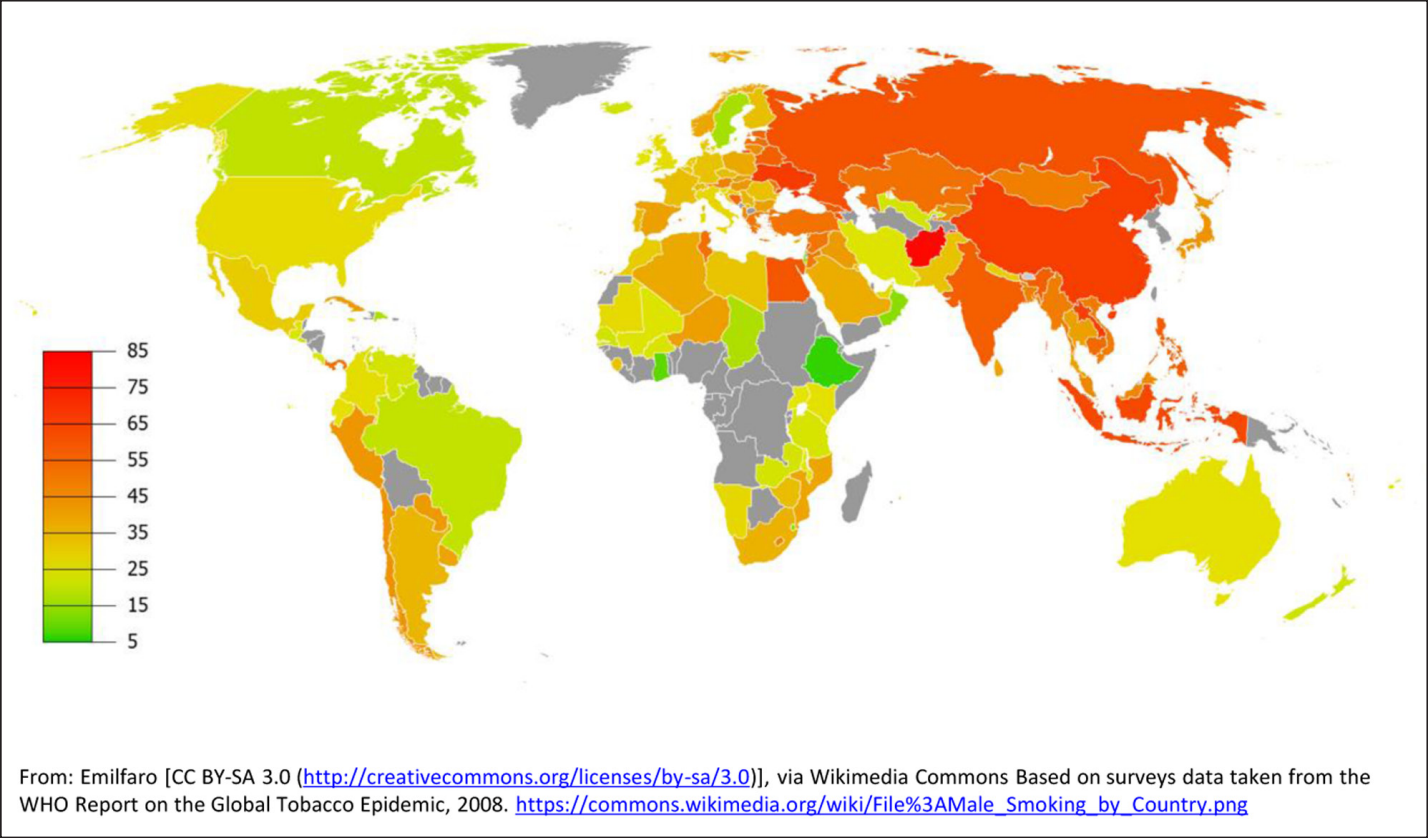
Global Smoking Prevalence in Males.

**Figure 2:**
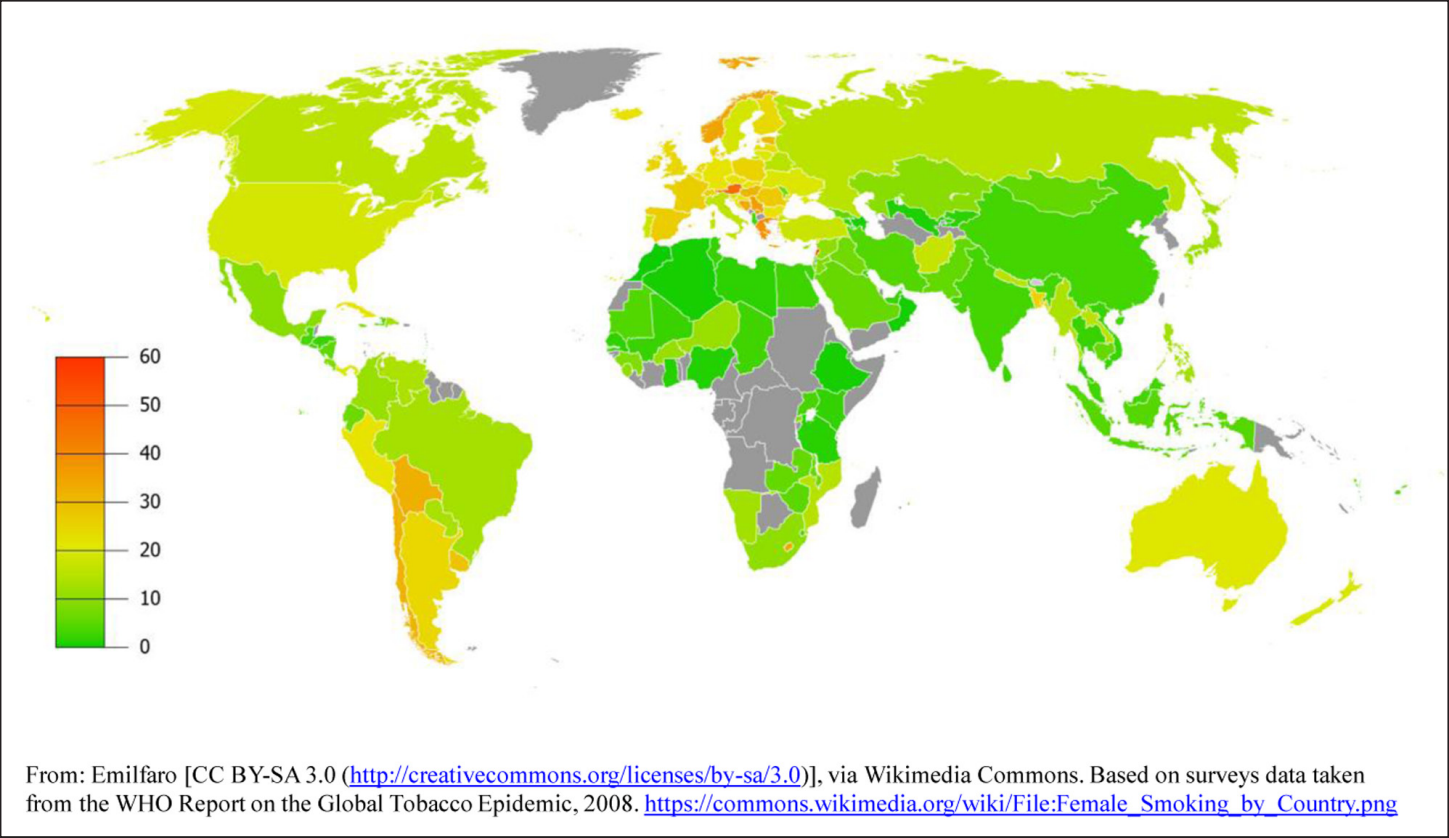
Global Smoking Prevalence in Females.

A 2008 WHO initiative conducted an analysis of the prevalence of tobacco use in Africa, the Americas, South-East Asia, Europe, the Eastern Mediterranean, and the Western Pacific [21]. The overall prevalence of smoking varied widely among the six WHO regions, ranking highest in Europe (29%) and lowest in Africa (8%). In general, men smoked more than women, with the largest disparities for daily cigarette smoking located in the Western Pacific Region, where men smoked 15 times more often than women, followed by South-East Asia where men smoked 10 times more often than women (Figures 1 and 2). Among men, the highest prevalence of smoking was in lower- to middle-income countries. Among women, relatively high rates of smoking (15%) were reported in upper-middle and high-income countries. Smoking rates were about five times lower in low- and lower-middle-income countries and in general, smoking prevalence declined as country per capita income rose.

## Developed Countries

Recent data from the National Health Interview Survey showed that the prevalence of smoking among United States (US) adults aged ≥18 years has declined from 20.9% in 2005 to 16.8% in 2014 [22]. Smoking continues to be more prevalent in males, adults aged 25–44 years, multiracial individuals, and American Indian/Alaska natives. It is also more common in individuals who have lower educational attainment, those living below the federal poverty level, those insured by Medicaid, the uninsured, and patients with disabilities [22]. Unfortunately, data from a study found a very high prevalence of sporadic tobacco use (57.3% to 67.4%) in young adults, a population that should be the target of anti-tobacco efforts [23]. That notwithstanding, the US has experienced a substantial reduction in the prevalence of cigarette smoking since the landmark 1964 Surgeon General’s Report [1], especially among white males. Much needs to be done since trends have changed relatively little among those with substance abuse or other psychiatric disorders, and smoking has even increased in some demographics, such as economically disadvantaged women [24].

Tobacco control in Great Britain has also improved in recent years. The prevalence of cigarette smoking was very high in the 1970s but declined rapidly (from 45% to 35%) in the early 1980s. The rate of decline then slowed, with smoking prevalence falling by only about one percentage point every two years until 1994, after which it stabilized at about 27% before resuming a slow decline in the first decade of the twenty-first century [25]. Recent data from the Opinions and Lifestyle Survey showed that overall smoking prevalence has continued to decrease in Great Britain. By 2012, 20% of adults (aged 16 years and over) continued smoking, a prevalence similar to previous years, but significantly lower than the 26% reported in 2002 [26]. The survey also showed that cigarette smoking in Britain is associated with marital status, with only 14% of the married population being active tobacco users. Smoking was far more common among single, unmarried individuals. Men reported a higher prevalence of smoking than women in all socio-economic strata with some exceptions (large employers and high managerial positions) [21].

Tobacco use is heterogeneous across Europe. Smoking is more common in Turkey, Latvia, and Greece, particularly among men. In Western Europe, Spain has a high rate of male smokers. The former Yugoslav Republic of Macedonia, Greece, Hungary, Poland, the Netherlands, and France have the highest rates of smoking among females, while Sweden is the only member of the European Union where more women smoke than men [27]. Policy initiatives in Europe addressing tobacco use have been historically limited. Recently, however, several countries – most notably Poland, Hungary and the Baltic states – have enacted tobacco legislation stricter than other European Union countries [28].

## Low- and Middle-income Countries

The Global Adult Tobacco Survey was designed to collect representative data on tobacco use in low-income and middle-income countries [29,30]. The study included national surveys conducted between 2008 and 2010 in 14 countries in Asia, America, and Europe [12]. Forty-eight percent of men and 11% of women smoked amid a population of 3 billion individuals. Tobacco consumption was quite variable, ranging from 21.6% of men in Brazil to 60.2% in Russia. Rates of cigarette smoking among women were also quite variable, with only 0.5% of women smoking in Egypt as compared to 24.4% in Poland. Women aged 55–64 years began smoking at an older age than did equivalently aged men in most countries. Younger men and women started smoking at similar ages. Smoking cessation rates were very low overall (<20%), particularly in China, India, Russia, Egypt, and Bangladesh. This large collaborative study reinforced the view that efforts to prevent smoking from becoming a habit and promote cessation of tobacco use are needed to reduce smoking related morbidity and mortality worldwide [12].

## Emerging Economies

China represents a daunting public health challenge since more than 300 million people smoke while an estimated 740 million, including 180 million children, are exposed to secondhand smoke [31]. Approximately one third of global tobacco leaf production is Chinese and 30% of all cigarettes are consumed in China. Smokers in China are mostly male, while only 2.4% of women smoke regularly compared with 63% of middle-aged men 45–64 years of age [32]. However, as many as 82% of women in China are exposed to secondhand smoke [33]. The prevalence of smoking is highest in rural areas and among the poorly educated. Since mass cigarette consumption is relatively new to China, the impact of the current epidemic cannot be overstated. It is poised to surpass that seen in western nations with an estimated one third of Chinese men likely to be killed by tobacco [34].

## The Negative Health Impact of Tobacco

Cigarettes are a complex mixture of highly addictive products: nicotine, polyaromatic hydrocarbons, Phenols, and nitrosamines, which are submicron-sized solid particles; other components, such as carbon monoxide, hydrogen cyanide, and nitrogen oxides, are gases. When a cigarette is smoked, there are approximately 600 ingredients that, when burned, create more than 7,000 chemicals, including at least 70 known carcinogens [35].

Overall, mortality is three to five times greater in smokers than in nonsmokers for both men and women. As a consequence, it has been recently estimated that smokers can lose more than 10 years of life expectancy [36,37]. Presently, the three major non-infectious causes of morbidity and mortality are cardiovascular diseases, chronic obstructive pulmonary disease (COPD), and lung cancer, all preventable causes of death [38,39,40].

Lung cancer is a well-recognized negative consequence of the tobacco epidemic. Approximately 80–90% of lung cancer cases are tobacco-related. The risk of lung cancer is greater among those who start smoking at a younger age or who smoke “high-yield” cigarettes, but it is also strongly correlated with the cumulative lifetime smoking exposure [39,40,41,42,43,44,45]. Although lung cancer was previously considered mostly a disease of males, since the 1950s lung cancer deaths among women have increased more than 600% [43]. As a consequence, lung cancer had surpassed breast cancer as the leading cause of cancer deaths in women in the late 1980s [44,45]. Unfortunately, the carcinogenic effect of tobacco is not limited to the lungs. Smoking generates circulating carcinogens, leading to increased risk for several other malignancies. Tobacco has been linked to 13 different types of cancer including breast [36,39,40,46,47], liver, pancreas, and colorectal tumors, among others [39,40,41,42].

COPD is a major public health problem and will remain a challenge for clinicians in the twenty-first century due to its high morbidity and mortality [48]. The main risk factor for COPD is exposure to tobacco smoke, either active or secondhand smoke. Other risk factors include exposure to indoor and outdoor air pollution and occupational dusts and chemicals [49]. Current projections estimate that by 2020, COPD will be the third leading cause of death worldwide, behind heart disease and cancer, and the fifth leading cause of years lost through early mortality or disability [50,51]. Cigarette smoking also significantly contributes to cardiovascular morbidity and mortality. Epidemiologic studies strongly support that cigarette smoking, in both men and women, increases the incidence of myocardial infarction and fatal coronary artery disease [52,53,59]. Smoking accounts for 33% of all deaths from cardiovascular disease and 20% of deaths from ischemic heart disease worldwide [47]. Smoking appears to have both causal relationships and possible synergistic interactions with other major risk factors for coronary heart disease, including hyperlipidemia, hypertension, and diabetes mellitus [54]. Importantly, nonsmokers exposed to secondhand tobacco smoke at home or work also have a 25–30% higher risk for heart disease [55,56,57].

Smoking and exposure to secondhand smoke are associated with primary tuberculosis (TB) infection, active disease, risk of recurrence, and TB mortality, with more than 20% of global TB incidence being attributed to tobacco [58]. Smokers are also at increased risk for erectile dysfunction, cataracts, periodontitis, gastro-esophageal reflux, and hip fractures [39]. Moreover, new studies implicate tobacco as a cause of adult onset diabetes, age-related macular degeneration, compromised immune system, and an increased risk for respiratory infections [39]. Women who smoke during pregnancy are more likely to have low birth weight and premature babies and increased risk for miscarriage [39]. There is now evidence linking smoking with ectopic pregnancy and orofacial cleft in newborns [36].

## Anti-smoking Legislation and Anti-tobacco Campaigns

Smoking is a global concern that demands international cooperation leading to the implementation of measures that will effectively control and ultimately put an end to the current epidemic. Decades have now elapsed since tobacco consumption was first linked to illness, particularly cancer. Since then, landmark anti-smoking campaigns emerged and gave rise to warnings about tobacco’s harmful effects on cigarette packs [67]. The WHO’s Framework Convention of Tobacco Control (FCTC) was the first international health treaty to arise from these efforts. It was endorsed by 180 countries across the globe, accounting for 90% of the world’s population (Table 1). The treaty aims to provide the necessary tools for promoting effective legislative changes for controlling tobacco consumption. Demand and supply were equally addressed by the treaty, including measures related to tobacco product pricing and taxation, education, and awareness. Much emphasis was placed on publicity and sponsorship of tobacco, reducing tobacco dependence as well as protecting passive smokers in public and work settings from the perils of secondhand smoking.

**Table 1:** World Health Organization Framework Convention on Tobacco Control Participating Countries.

Europe	North America	Central America	South America	Asia/Middle East	Africa	Oceania

Albania	Canada	Costa Rica	Argentina	Afghanistan	Algeria	Australia
Austria	United States	Cuba	Bolivia	Azerbaijan	Angola	New Zealand
Belgium		Dominican Republic	Brazil	Bangladesh	Botswana	
Bosnia		El Salvador	Chile	Belarus	Burkina Faso	
Bulgaria		Guatemala	Colombia	Cambodia	Burundi	
Croatia		Haiti	Ecuador	China	Cameroon	
Czech Republic		Honduras	Guyana	India	Central African	
Denmark		Mexico	Paraguay	Indonesia	Republic	
Estonia		Nicaragua	Peru	Iran	Chad	
Finland			Uruguay	Iraq	Congo	
France				Israel	Ivory Coast	
Germany				Japan	Egypt	
Greece				Kazakhstan	Ethiopia	
Hungary				Korea	Gabon	
Ireland				Lebanon	Gambia	
Iceland				Malaysia	Ghana	
Italy				Mongolia	Guinea	
Latvia				Nepal	Kenya	
Lithuania				Oman	Liberia	
Norway				Pakistan	Libya	
The Netherlands				Philippines	Madagascar	
Poland				Qatar	Mali	
Portugal				Russian Federation	Morocco	
Romania				Saudi Arabia	Mauritania	
Slovenia				Thailand	Mozambique	
Spain				Turkey	Namibia	
Switzerland				Turkmenistan	Niger	
United Kingdom				United Arab	Nigeria	
Ukraine				Emirates	Ruanda	
				Uzbekistan	Senegal	
				Vietnam	Swaziland	
				Yemen	Tanzania	
					Tunis	
					Uganda	
					Zambia	
					Zimbabwe	

Tax increases constitute one of the most effective means of controlling smoking by decreasing demand for cigarettes. Arguably, controlling tobacco prices through taxation reduces the number of premature deaths linked to tobacco consumption and mitigates the costs by increasing revenue. The money can be used to sponsor anti-smoking initiatives and shoulder the health costs. Unfortunately, only 33 countries (10% of the world’s population) have levied taxes amounting to more than 75% of the product’s value. Similarly, only 29 countries have banned any form of tobacco advertising, while one third of treaty members either completely lack or barely offer minimum restrictions. Illicit commerce is also a growing problem, since approximately one out of ten cigarettes and other tobacco products eschew regulation and taxation [60]. The interdiction of such commerce will contribute to minimize the consumption of tobacco by limiting the existence of more affordable alternatives to legally purchased cigarettes, resulting in an increase of tobacco cost, which historically limits their use.

Implementation of anti-smoking legislation has led to large reductions in exposure to secondhand smoke. In Scotland, the effect has been particularly impressive for nonsmokers living in nonsmoking households with mean cotinine concentrations falling by 49% [61]. Unfortunately, non-smokers living in smoking households had a negligible reduction. Smoke-free housing policies may be an effective strategy for reducing secondhand exposure in these individuals [62]. A recent meta-analysis confirmed that anti-smoking legislation implemented between 1991 and 2010 has led to a 12% reduction in hospitalizations for acute coronary events worldwide [63]. A different meta-analysis published in 2012 also confirmed that anti-smoking legislation in 33 countries reduced the risk of smoking-related illnesses, including cardiac, cerebrovascular, and respiratory diseases. In both studies, more comprehensive laws were associated with greater risk reductions and long-term benefits [64].

The need for comprehensive legislation cannot be overstated. In Spain, an initial attempt to protect workers from secondhand smoke that allowed workplaces to designate smoking areas was only partially effective. For nonsmoking employees of bars and restaurants where smoking was allowed, exposure to secondhand smoke remained similar to pre-legislation levels. Conversely, cotinine levels decreased by 56% in workplaces where smoking was totally prohibited [65]. Internal tobacco industry documents made public through US litigation settlements show that tobacco companies have promoted separate seating for smokers and ineffective ventilation technologies [66]. Fortunately, continued efforts have led to more comprehensive legislation. For example, the original Spanish anti-tobacco law approved in 2005 was amended in 2010. Overall reductions in exposure to secondhand smoke reached 22% between 2005 and 2007 followed by an additional 17% between 2007 and 2011 in response to the new legislation [67]. Data shows that special emphasis must be placed on young individuals and frequently overlooked tobacco exposure settings [68]. In Italy, for example, despite comprehensive legislation, secondhand smoke exposure remained a comparatively high 54% in those aged 15–24 years, primarily due to exposure inside motor vehicles [69]. A US study showed that self-reporting is an unreliable source of secondhand exposure [70]. In this study, 31% and 53% of subjects with measurable cotinine and nicotine levels did not report exposure, suggesting that efforts by the smokers’ lobby to categorize current legislation as exaggerated or overreaching may be misguided or unfounded.

It is legitimate, however, to consider the limits of comprehensive legislation enacted to protect those exposed to cigarette smoke. A symposium convened by the Tobacco Control Legal Consortium at William Mitchell College of Law in 2007 is illustrative of the hurdles we face in advancing anti-smoking legislation in the public arena [71]. Participants expressed concerns about the implementation of zero-tolerance legislation for fear that it might undermine the scientific credibility of evidence-based tobacco control policies. The authority of employers to regulate off-site smoking of their employees was also questioned. On the contrary, evidence was cited supporting smoking bans in vehicles, especially for the sake of small children. However, anti-tobacco legislation can have unanticipated negative consequences. Variable enforcement of a ban on smoking in parks and beaches in Vancouver, Canada, for example, led to higher smoking rates in poorer neighborhoods, generating health inequities [72].

Children, arguably the most vulnerable age group and the focus of many efforts to curb cigarette smoking, continue to be exposed despite current legislation and voluntary bans on smoking. A systematic review of the literature showed that children of smoking parents are up to 13 times more likely to be exposed to secondhand smoke [73]. Studies such as these suggest that established measures may be reaching a point of diminishing returns, raising interest in alternative ‘endgame strategies’, such as limiting nicotine levels, raising the pH of inhaled smoke, driving prices upward, or even setting a cutoff banning smoking for young adults altogether [74]. While such measures are often advocated, experience with alcohol prohibition has shown that illicit commerce will limit the impact of such strategies.

## Conclusions

While the devastating impact smoking has on global health is well accepted, continued efforts to curb its prevalence face ongoing challenges. Recent legislation, especially in developed nations, has demonstrated the benefit of tobacco bans implemented for the protection of secondhand smokers in public spaces, and ongoing efforts are investigating the limits of the law in curbing tobacco use in private spaces or shared outdoor venues. Much remains to be done as the tobacco epidemic continues unabated in some regions of the world, and as we brace for the imminent public health impact of smoking in low- and middle-income countries that have only recently engaged in mass tobacco consumption.
